# 3D Printing of Fibre-Reinforced Thermoplastic Composites Using Fused Filament Fabrication—A Review

**DOI:** 10.3390/polym12102188

**Published:** 2020-09-24

**Authors:** Andrew N. Dickson, Hisham M. Abourayana, Denis P. Dowling

**Affiliations:** School of Mechanical and Materials Engineering, University College Dublin, Belfield, D04 V1W8 Dublin, Ireland; hisham.abourayana@ucdconnect.ie (H.M.A.); denis.dowling@ucd.ie (D.P.D.)

**Keywords:** fused filament fabrication, polymers, fibre reinforcement, mechanical properties

## Abstract

Three-dimensional (3D) printing has been successfully applied for the fabrication of polymer components ranging from prototypes to final products. An issue, however, is that the resulting 3D printed parts exhibit inferior mechanical performance to parts fabricated using conventional polymer processing technologies, such as compression moulding. The addition of fibres and other materials into the polymer matrix to form a composite can yield a significant enhancement in the structural strength of printed polymer parts. This review focuses on the enhanced mechanical performance obtained through the printing of fibre-reinforced polymer composites, using the fused filament fabrication (FFF) 3D printing technique. The uses of both short and continuous fibre-reinforced polymer composites are reviewed. Finally, examples of some applications of FFF printed polymer composites using robotic processes are highlighted.

## 1. Introduction

**Figure 1:**
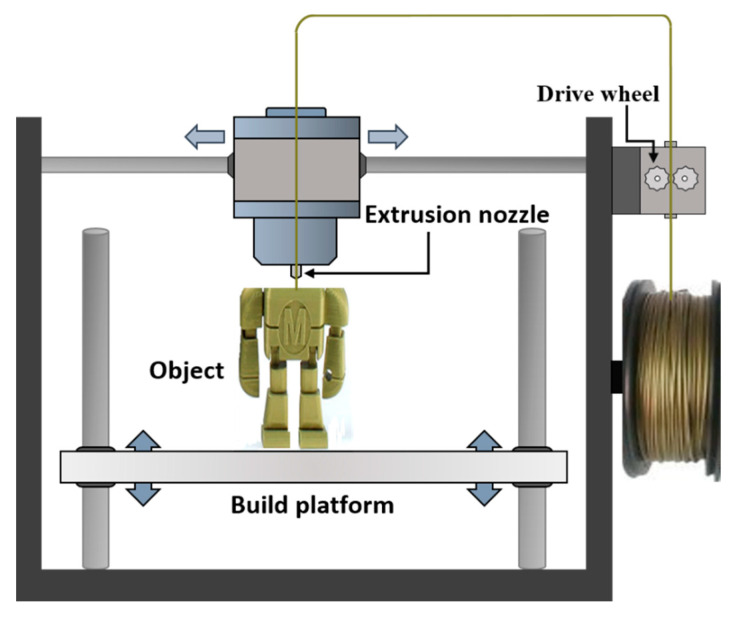
Schematic of FFF process for the printing of parts using the melted polymer filament.

Three-dimensional (3D) printing, also known as additive manufacturing (AM), can be used to print a range of metallic, polymer and composite parts with complex geometries and great design flexibility, while minimising processing waste [1,2]. Applications of this processing technology have included parts fabricated for use in the biomedical, automotive and aerospace sectors [3]. Three-dimensional printing was first introduced during the early 1980s, using the process of stereolithography, in which UV lasers are used to cure layers of polymer into 3D shapes [4]. These methods can be used to process materials such as epoxies. For example, a hydrogel combined with a UV curable adhesive to form a composite exhibiting properties similar to organic tissues, such as cartilage, was demonstrated [5]. A range of other polymer 3D printing technologies are also available, including Selective Laser Sintering (SLS), Laminated Object Modelling (LOM), Multi Jet Fusion Printing and Fused Filament Fabrication (FFF) processes [6,7,8]. The latter technique, which is also known by the trade name Fused Deposition Modelling, is one of the most widely used amongst all the 3D printing techniques, showing great potential for fabricating 3D geometry parts with the capacity to compete with conventional processing techniques [9,10,11]. In this technique, the polymer filament is extruded through the nozzle that traces the part’s cross sectional geometry layer by layer, as shown in Figure 1 [12]. The nozzle contains resistive heaters that keep the polymer at a temperature just above its melting point, so that it flows easily through the nozzle and forms the layer [13]. The extruding apparatus is typically mounted onto an X–Y computer numerical control (CNC) gantry, allowing the printing of complex geometric patterns. Once a pattern is deposited, the build platform is lowered, or the extruding orifice is raised-up, to deposit the next layer [14,15].

At present, thermoplastic polymers are the most frequently utilised feed-stock materials for the FFF process, due to their relatively low cost as well as their low melting temperatures [16]. These polymers include acrylonitrile butadiene styrene (ABS), polylactic acid (PLA), polycarbonate (PC), polyether ether ketone (PEEK) and nylon [17]. The resulting pure polymer products, however, can often lack the strength to produce fully functioning engineering parts, which has restricted the wider adoption of this technology [18]. In order to address this issue, reinforcing materials, such as fibres, are added into the polymer matrix during printing, in order to produce a composite structure which typically exhibits improved mechanical properties [19].

Reinforcing fibres in composite materials can be in the form of continuous fibres or discontinuous (short) fibres [20]. Continuous fibres have long aspect ratios and have a preferred orientation, while short fibres have short aspect ratios and generally have a random orientation [21]. Due to the fibre orientation, continuous fibre composites offer higher strength and stiffness qualities than those of discontinuous fibre composite [22]. This paper will initially introduce and discuss short fibre-reinforced polymer composites, before reviewing the rapidly expanding field of 3D printed continuous fibre composites. Commercial developments of FFF, including the use of robotic printing techniques for larger scale printing, are also reviewed.

## 2. 3D Printing of Short Fibre-Reinforced Composite

Composites fabricated reinforced using short fibres are attractive because of their ease of fabrication, economy and superior mechanical properties [23]. They are typically produced by extrusion compounding, injection, or compression moulding processes [22]. For FFF processing, the filaments are fabricated in a two-step process; this firstly involves mixing the polymer pellets and fibre in a blender and secondly extruding the compound to create the filament [24]. Typical short fibres used as reinforcements include carbon and glass fibres. Recently, basalt fibre has also received attention [25].

As detailed in Table 1, several authors have investigated the addition of short fibres into a thermoplastic polymer to provide composite filaments used as a feedstock for FDM process. The reported studies included investigations of the effect of fibre content, as well as its orientation and length, on the processability and performance of the resultant fibre-reinforced composites. Some studies involved a comparison between the properties of 3D printed composites and those fabricated by traditional compression moulding techniques.

Fibre content plays an important role in determining the properties of FFF composite filaments. Generally, tensile strength increases with increasing fibre content. Composite filaments, however, with high fibre content, can be very difficult to print, due to issues with nozzle clogging, in addition to the excessive viscosity of the melted composite filament [34,35]. Therefore, the determination of an appropriate fibre content in the composite used as a filament for FFF is often a compromise between processing difficulty and the performance characteristics of the resulting composites [32].

Ning et al. [36] investigated the effect of carbon fibre content and length on the mechanical properties and porosity of FFF printed ABS/carbon fibre composite parts. The composite filaments were fabricated with different fibre contents (3 to 15 wt %) and different fibre lengths (100 and 150 µm). This study demonstrated that the best performing FFF printed parts were obtained for samples reinforced with 5 wt % carbon fibre, which achieved 22.5% and 30.5% increases in tensile strength and Young’s modulus, respectively, compared with the ABS only specimens. A further increase in the fibre content to 10% or higher resulted in a decrease in tensile strength due to the higher porosity. Moreover, the composite specimens reinforced with longer carbon fibres (150 μm) exhibited higher tensile strength and Young’s modulus values, and lower toughness as well as ductility, compared with those reinforced with shorter carbon fibres (100 μm).

**Figure 2:**
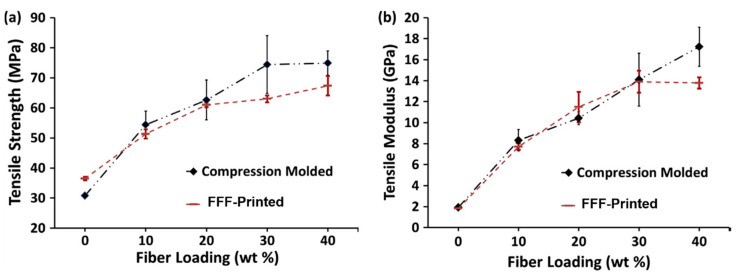
Effect of fibre content and preparation process on (**a**) tensile strength and (**b**) modulus of ABS/carbon fibre composites [34].

A study by Tekinalp et al. [34] investigated short fibre (10–40 wt %)-reinforced ABS composites as a feedstock for FFF printing, in order to report on their processability, microstructure and mechanical performance. The additive components were also compared with traditional compression moulded (CM) composites. The results showed that FFF 3D printing yielded samples with very high fibre orientation, lower average fibre length and high porosity levels (16–27%) compared with those obtained using the CM process. Tensile test results demonstrated that tensile strength and modulus were increased, with increasing fibre content for both the FFF and compression moulded samples (Figure 2). However, the improvement in the mechanical properties of FFF 3D printed samples is close to that of those fabricated with CM process, attributed to the high degree of fibre alignment in FFF 3D printed samples compared to the random orientation of the fibres in moulded samples. The authors compensated for some of the loss of strength due to high porosity and decreased fibre length.

For load bearing applications, the composite filaments used in the FFF process must exhibit adequate mechanical properties, such as strength, stiffness, ductility and flexibility [19,32]. The addition of short fibres into pure thermoplastics polymers, however, while improving the resulting printed parts’ tensile and flexural strengths, can be at the cost of the reduced flexibility and handleability of the resulted composite filaments. Authors have addressed this issue through the addition of a small amount of plasticiser and compatibility agents. For example, the processability values of short glass fibre-reinforced ABS composites with three different glass fibre contents (10.2, 13.2 and 18 wt %) were investigated in relation to their use as a feedstock filament for FFF [32]. The ABS was mixed with glass fibre in a twin-screw extruder, and then granulated into small pellets. These pellets were then fed into a single screw extruder and extruded into a filament. The glass fibres were found to reduce the flexibility of the resulting filament and make it impossible to feed into the FFF printer, therefore plasticiser (linear low-density polyethylene) and compatibiliser (hydrogenated Buna-N) were added to improve the ability to process the filaments through the printer nozzle and the properties of the FFF parts. The properties of the resulting composite filaments showed they would work well as a feedstock for FFF processes. This study also demonstrated that adding short glass fibre to ABS polymer resulted in a reduction in adhesive strength between the layers in the resulting FFF 3D printed samples, while the tensile strength was increased with increasing fibre contents. The authors reported that this may be due to enhanced fibre bridging across layers during printing, as fibre content increased.

**Figure 3:**
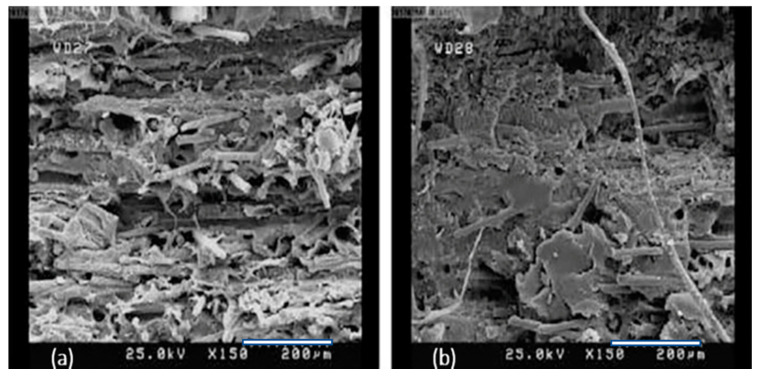
Scanning electron microscopy (SEM) images of the specimens manufactured using (**a**) 3D printing and (**b**) CM methods [33]. These images help to illustrate the differences in interlayer adhesion of 3D printed samples compared to that prepared using CM process.

A study by Sodeifian et al. [33] reported on how the flexibility of glass fibre-reinforced polypropylene composite filaments was enhanced by adding maleic anhydride polyolefin (POE-g-MA) as a modifier. POE-g-MA was added with three different weight percentages, namely 10, 20 and 30 wt %. The filaments were used to produce test specimens using FFF printing. The test specimens were also provided using the CM method, to compare the results with those of the FFF method. This study demonstrated that the tensile strengths of the specimens with 10 wt % POE-g-MA were the same irrespective of the manufacturing method used, but the FFF 3D printed specimens exhibited higher flexibility. The FFF 3D printed specimen with 20 wt % POE-g-MA showed superior mechanical properties, compared with those prepared by using the CM method. Increasing POE-g-MA to 30 wt % yielded an increase in the strength and a decrease in flexibility. X-ray diffraction analysis indicated the higher crystallinity of the specimens prepared by compression moulding, compared with that obtained using 3D printing. Figure 3 helps to demonstrate the interlayer and intralayer adhesion of the 3D printed specimens, as compared to that prepared using the CM method.

A study by Sang et al. [35] investigated PLA composites reinforced with silane treated basalt fibre at three different fibre weight fractions (5, 10 and 20 wt %). The effects of fibre type, as well as its weight fraction, on the thermal properties, mechanical performance and rheological behaviour of PLA/BF composite filaments were investigated. The study included a comparison with composites fabricated using both compression-moulded and carbon fibre-reinforced counterparts with the same fibre weight fraction. The results of tensile tests indicated that 3D printed specimens exhibit similar tensile strength values to the compression moulded specimens, and this was attributed to the high alignment of fibre orientation in FFF 3D printed samples versus the random orientation of fibres in the moulded samples. A 33% increase in tensile strength was obtained for FFF samples reinforced with 20 wt % basalt fibre. Rheological results, as anticipated, demonstrated that the viscosity of the carbon fibre composites is higher than that of the PLA matrix, while that of the basalt fibre composite counterparts is close to the PLA only material. The high viscosity of the carbon fibre composite results in poor printing flowability and interlayer bonding defects, which causes stress concentration and failures in test samples. This study indicates the ease of processability of basalt compared to carbon fibre for the fabrication of FFF composites.

### Applications of FFF Printed Short Fibre-Reinforced Composites

**Figure 4:**
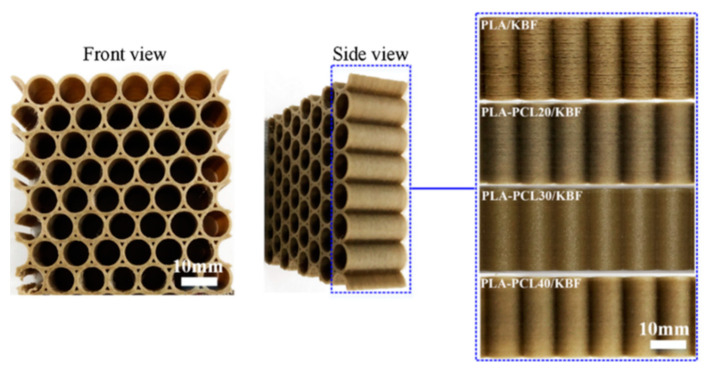
3D printed circular honeycombs of PLA-PCL/KBF with varying ratios [38]. The PLA-PCL/KBF composite consists of polylactic acid (PLA) as the stiff matrix, polycaprolactone (PCL) as an elastomer phase and silane-treated basalt fibres (KBF) as the reinforcing filler.

Some examples of the applications of fibre-reinforced polymer composites include high-temperature inlet guide vanes (IGV) for aerospace applications, printed using polyetherimides- Ultem 1000 mixed with 10% chopped carbon fibre [19,37]. Sang et al. [38] developed promising PLA-PCL/basalt fibre composite filaments, to be used as FFF feedstock for manufacturing honeycomb structures that exhibit elastic deformations with superior energy absorption under compressive loading, and which provide valuable means to obtain an excellent compressive mechanical performance in honeycomb structures (Figure 4).

## 3. 3D Printing of Continuous Fibre-Reinforced Composite

As discussed in Section 2, the incorporation of short fibre reinforcement can usually increase the stiffness of the resulting composite; however, the part strength is often only marginally increased. This is due to reliance on the matrix material to transfer loads between fibres. In contrast, a continuous fibre reinforcement transfers and retains primary loads within unbroken strands of fibre, and this results in a significantly lower load transfer through the polymer and allows for a load-bearing capacity orders of magnitude higher than that which short reinforcement is capable of achieving. In the case of continuous fibre composites, the polymer serves to transfer off-axis loads between fibres, such as shear forces. This protects the fibres, as high modulus reinforcement such as carbon and glass fibre exhibit poor mechanical properties under shear loading [39]. Continuous fibre-reinforced polymers are currently one of the largest areas of focus in 3D printing research [40]; this is due to their potential to match or exceed the mechanical performance of conventional composites. A number of authors have reported on modifications of the standard FFF process for the printing of continuous fibres; these include hardware changes, such as more wear-resistant nozzle materials, fibre cutting mechanisms, dual inlet hot-ends and fibre preheaters [41,42].

**Figure 5:**
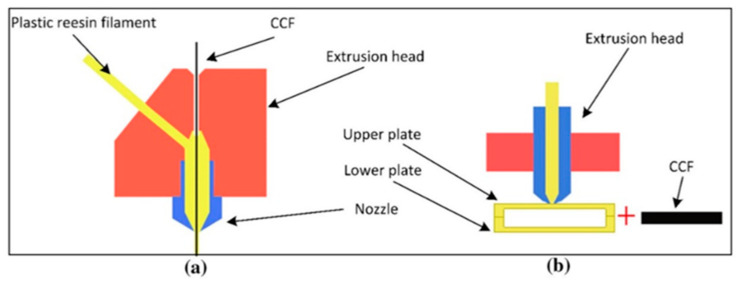
Schematics of in-situ fusion techniques: (**a**) in-nozzle impregnation with polymer and coaxial fibre extrusion, and (**b**) embedding of continuous carbon fibre (CCF) after 3D printing in a thermal bonding process. Images from [42].

Two main categories of continuous fibre printing have been described in the literature, these being “in-situ fusion”, and “ex-situ prepreg” [41,42]. The in-situ systems utilise two input materials, typically a dry fibre feedstock (the reinforcing fibre) and a neat polymer (the matrix polymer), which are combined together during the printing process. One of the most widely used techniques is known as “in-nozzle impregnation” [43]. In this process, the dry fibre is typically pre-threaded through the printer nozzle prior to printing, and the fibre is also preheated using a coil heater or IR lamps, so as not to excessively cool the molten polymer during printing. The polymer is fed by a motor-driven hobbed gear into the melt zone of the hot-end, and the preheated fibre and melted polymer converge in this melt zone where they are pushed together by the feeding polymer filament. The polymer continues to flow as long as the motor drives the filament, and the continuous fibre bundle is pulled through the nozzle by traction force as it is anchored to the build plate, as shown in Figure 5a. This method has the advantage of a single manufacturing step, which uses low-cost commercially available feedstocks, such as carbon fibre tow and FFF filaments. It also allows for real-time control over the local volume fraction of the part by altering the flowrate of polymer. This single-step printing approach, while rapid, has been reported to yield relatively poor-quality composite parts [44,45]. The short dwell time within the heated nozzle results in poor polymer infusion into the fibre bundles, and ultimately increases the porosity of the composite [43,44,45]. Tian et al. [45] reported that increasing the printing temperature led to a marked increase in polymer impregnation and decreased porosity, however a quantitative analysis was not performed. Despite these issues, these studies have demonstrated that significant strength increases can be achieved by the addition of the continuous fibre to polymer materials. Matsuzaki et al. [46] reported on a 3.4-fold improvement in the strength of carbon fibre-reinforced PLA, versus unreinforced polymer, when only 6.6 volume fibre percent (VF%) was used. Similarly, Bettini et al. [47] observed up to a six-fold improvement in strength with the incorporation of 8.6 VF% of aramid fibre in PLA. This aramid composite was shown to exhibit a lower porosity compared with that obtained for carbon fibre composites. Another “in-situ fusion” method involves first printing polymer parts using standard FFF 3D printing techniques; these are then sandwiched together with a composite reinforcement and bonded with the application of heat and/or pressure [48]. The fibres can be added during the printing process and overprinted or added after printing between layers of the printed polymer, and then placed into an oven to facilitate bonding (Figure 5b). Mori et al. [48] developed a method for overprinting and compared it with the in-nozzle impregnation method discussed earlier. Whilst overprinted carbon fibre/PLA samples led to a 180% increase in load before failure versus the unreinforced PLA, the in-nozzle impregnated composite withstood a force of 500%, which was obtained using PLA only. This superior performance was attributed to the better contact between the matrix and fibre, and as a result significantly reduced porosity. A case study by Brooks et al. [49] used a topologically optimised FFF polymer base structure, onto which large fibre tows were adhesively bonded, and the results were a lighter part that exhibited a 4000% higher strength, compared with the unreinforced equivalent. A potential weakness of these processes is the short amount of time the fibre spends in the molten polymer, as well as the low pressure applied to the polymer, which typically leads to poor infiltration and high porosity. The approach also necessitates that multiple manufacturing steps occur simultaneously, which makes optimisation difficult.

**Figure 6:**
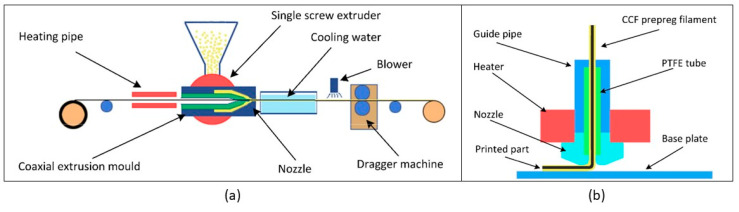
Schematic of ex-situ prepreg process: (**a**) The extrusion and cooling apparatus for production of the prepreg filament. (**b**) The printing process utilising the prepreg filament requires no drive gear as the fibre is pulled through the nozzle, extruding the polymer as it moves [42].

In contrast with “in-situ fusion”, the “ex-situ prepreg” systems separate the manufacturing of the filament and the printing of the composite into two separate steps. This allows for greater control over the individual processes. As with in-situ systems, the method utilises two input materials (a fibre tow and polymer); however, these are combined together prior to printing into a pre-impregnated filament (prepreg), via a separate extrusion process (Figure 6). The prepreg filament is then spooled and transferred to the printing system for deposition. Where in-nozzle impregnation requires a drive motor for the constant extrusion of the polymer, this method only requires a motor to feed the initial few millimetres of filament through the nozzle. After the filament is anchored to the plate, this motor disengages and is pulled through the nozzle by the continuous fibre reinforcement, which remains solid throughout the process. This simplifies the printing process significantly, and as well as allowing for superior fibre impregnation during the filament manufacture stage, the dedicated extrusion apparatus can exert more pressure on the polymer to fully infuse the fibre tow, and allows for higher manufacturing speeds.

In 2014, the MIT spin-out company Markforged was the first to commercially offer the ex-situ prepreg composite printing system [50]. Their system could print using carbon, glass and Kevlar fibre, and used a cutting apparatus to deposit the correct amounts of fibre in specific locations in the part. The printer included a second FFF printhead for the printing of unreinforced nylon. Selected regions of a polymer part can be reinforced, rather than strengthening the entirety of the component. In doing so the components’ fibre fractions are limited to approximately 34 VF% (slightly lower than the VF% of the prepreg filament used), and large areas of the parts remain unreinforced [41]. Compared with printed polymer only parts, the FFF of the composites exhibited significantly improved mechanical performances. For example, Blok et al. [51] reported tensile strengths as high as 725 MPa for carbon fibre/PA, compared with 84 MPa for the printed PA polymer. It is important to highlight, however, that the results from this study are based upon samples that were modified after printing. Several studies utilise an in-house designed filament or printer with a similar mechanism to that used by Markforged. Hu et al. [42], for example, produced a custom PLA/carbon fibre prepreg filament printed using a modified open source 3D printer, and these composites achieved flexural strengths five times higher than unreinforced PLA. However, the VF% was not provided for cross comparisons to be made. The air void content of these samples is typically lower than in the in-nozzle impregnated equivalents, primarily due to the initial impregnation step. As highlighted by Matsuzaki et al. [52], increasing the fibre count in a tow results in an increased air content, however after deposition this is typically reduced. This study also demonstrated that after printing under pressure (from the printing head) the nozzle serving to push the air out of the filament, a reduction in porosity from 33% to 4% was observed for some samples [52]. Whilst this result was reported for a single line of printed filament, the overall composite’s void content increased due to the formation of air pockets between filaments and subsequent layers, as they are placed adjacent to and on top of one another.

Both “in-situ fusion” and “ex-situ prepreg” systems demonstrate that entrained air within the composite matrix is the primary challenge when 3D printing continuous fibre composites. Prepreg systems have the advantage of a dedicated manufacturing step, which can reduce filament air content to nearly 0%. However, some level of porosity remains after printing, and it is therefore evident that the printing process itself induces porosity and still requires optimisation. Goh et al. [41] observed that the overlapping of fibre bundles can reduce this porosity, however it could not be eliminated completely. The reduction in printed part porosity associated with the use of low pressure processing conditions during printing (1 Pa) has been successfully shown to increase the interlaminar shear strength (ILSS) of carbon, glass and Kevlar, by 33%, 22% and 12% respectively, compared to those materials printed under atmospheric pressure [53]. Another method of both decreasing the porosity of the FFF printed parts, as well as enhancing interlayer adhesion, is the use of atmospheric plasma surface activation treatments. This was demonstrated through the use of an in-line air atmospheric plasma jet treatment for the activation of sized basalt fibres, immediately prior to the application of polypropylene by extrusion coating, to form the polymer-coated filaments [25]. The flexural modulus and the maximum shear stress values of the resulting FFF composites were found to increase by 12% and 13%, respectively, compared with those obtained for composites fabricated using untreated fibres. It was concluded that this increased mechanical performance is likely due to the enhanced interfacial bond strength between the fibres and the polypropylene polymer, with an associated reduction in the level of air incorporation around the basalt filaments.

### 3.1. 3D Printed Composites—Mechanical Performance Comparison

**Figure 7:**
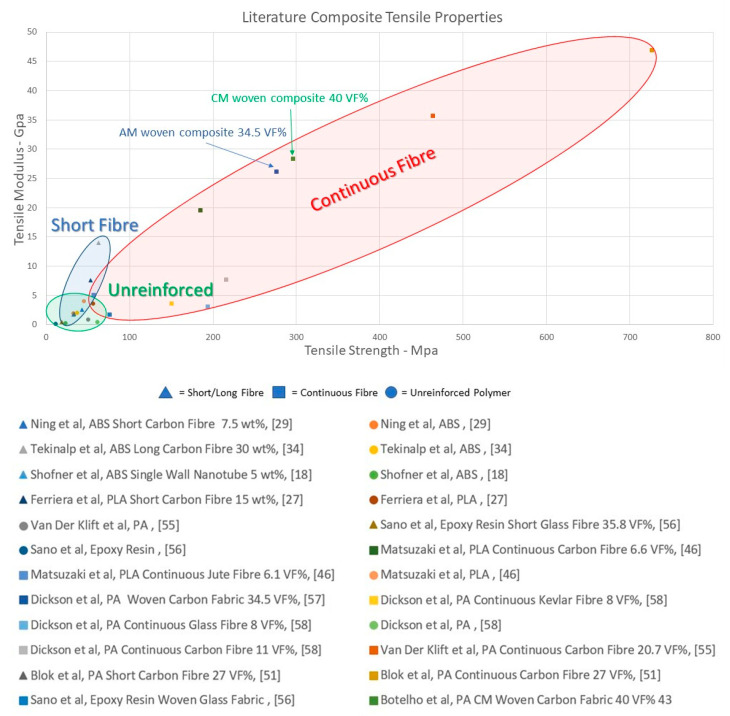
Literature values for tensile strength and modulus for short and continuous fibre-reinforced composites, as well as unreinforced polymers for comparison. A comparison between similar additive manufactured (AM) and compression moulded (CM) woven PA66/CF composites is highlighted, with tensile performance being comparable. Key: Author, matrix, reinforcement, fibre % [18,27,29,34,43,46,51,55,56,57,58].

Agarwal et al. [54] demonstrated how the 3D printed composites can outperform those produced using conventional approaches, particularly when the printing is carried out using optimised fibre orientations. Figure 7 provides an overview of the tensile properties of continuous reinforcement composites versus short, particle and unreinforced polymers, based on values reported by a number of authors in the literature. The wide range of tensile properties obtained for a given composite type is likely to be influenced by parameters such as fibre content, as well as composite processing technique used.

### 3.2. Continuous Fibre Printing—Pathing Behaviour

#### 3.2.1. Open Source Programme

**Figure 8:**
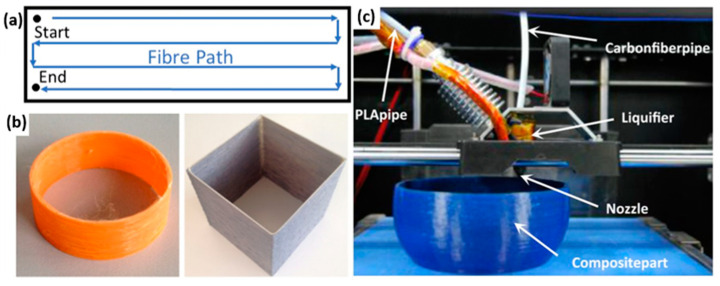
(**a**) Example of a raster pattern generated for printing tensile testing samples [41]. (**b**) Objects printed using “spiral” generated by an FFF slicer software [47]. (**c**) Printer producing a bowl-shaped component from PLA/CF [45].

Printing parameters such as temperature, speed, nozzle height, cornering radii and filament overlap can have an impact on the printed composite’s mechanical performance. As the majority of composite printing systems under development are based upon a 3-Axis CNC (Computer numerical control) platform, they utilise Gcode to control movements. Toolpath logic and printing behaviour must accommodate the unique behaviour of a filament, containing a continuous unbroken reinforcement. As the fibre reinforcement remains in a solid state throughout the printing process, its mechanical properties should be unaffected by the printing process, however this assumes that no destructive printing behaviour has occurred during deposition. “Destructive printing behavior” refers to any movement or printing parameter that results in a reduction of the final properties of the composite, compared with those of the pre-deposition material (such as porosity or fibre discontinuity). FFF slicing software (GrabCAD, Ultimakers CURA, Slic3r, simplify3D etc.), has developed significantly over the last 30 years, since its invention in 1989 by Scott Crump, founder of Stratasys Ltd. [59]. The majority of studies performed on custom-built continuous composite systems use a simple raster deposition pattern, which is a back and forth movement separated by raster gaps [42,44,45,46], as shown in Figure 8a. As raster patterns can be easily programmed using existing FFF software, or with milling CNC software, they allow for the creation of rectangular specimens in a short period of time. This is likely the reason for many studies focusing on tensile and flexural testing, as samples can be manufactured without complex pathing software. The tight corners of 180° introduce major fibre damage and dislocation during printing, however the attachment of tabs during testing usually obscures this fact [52]. Another popular printing method is to use FFF software perimeter-following logic to form rings of material in a spiral like motion. This mode can mitigate most of the problems of a raster pattern by taking corners at reduced angles [47]. It can be used to make simple geometries, such as cylinders, wing cross-sections or dog bones, or any solid shape that contains no internal features (i.e., no hollow spaces) (see Figure 8b,c). The primary reason behind the use of these patterns is that the toolpath generated is continuous, as most custom-built systems do not include a cutting apparatus, which is necessary for the printing of more geometrically complex shapes.

#### 3.2.2. Proprietary/Commercial Programs

**Figure 9:**
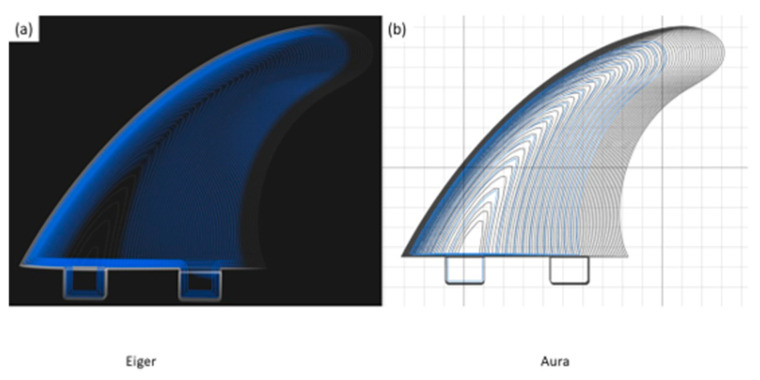
Comparison of (**a**) Markforged “Eiger” and (**b**) Anisoprint “Aura” slicer software for fibre composite printing. The Eiger software facilitates fibre placement in tighter spaces, with blue lines indicating fibre paths.

In order to facilitate the commercialisation of the 3D printing technology for composite production, several companies have simplified the tool pathing software and aligned the method of operation more closely with that of FFF systems. Between 2014 and 2019, only one such software was available commercially; this was the Markforged slicer software “Eiger”. This software allows for automated fibre placement based upon existing slicer logic, such as perimeter placement, but can only be utilised with Markforged Mark series composite printers [60]. As of 2019, additional companies have released open sourced equivalents of slicing software, with a specific focus on turning FFF printers into composite printers. For example, 9T labs of Zurich, Switzerland and Anisoprint of Moscow, Russia have both released open sourced slicer software for use on a wide range of 3-axis printing systems [61,62]. 9T labs is in early beta testing of its “CarbonKit” and accompanying slicing software; this system serves as a drop-in kit for existing FFF printers, expanding their capabilities into composite printing. The accompanying software “Fibrify” appears similar in function to Markforged “Eiger”, with a greater emphasis on fibre optimisation. Anisoprint has followed a similar route to that of Markforged with its “Aura” slicer, which enables fibre printing with their desktop “Composer” series printers [61,62]. As with “Eiger” and “Fibrify”, this slicer is fundamentally an FFF slicer, with the added functionality of fibre inclusion. These commercial systems cater to a model of “improving printed part performance” rather than “improving composite part performance”. Figure 9 compares the Markforged “Eiger” and Anisoprint “Aura” slicer software for fibre composite printing. As demonstrated in this literature review, the inclusion of fibres will ultimately lead to an increase in the strength and stiffness of FFF parts, however the performance of the resulting composite part can be significantly enhanced by optimising the fibre placement.

### 3.3. Geometrically Complex Composite Fabrication through 3D Printing

The majority of the studies to date on composites have focused on improving their mechanical properties. 3D printing systems, however, also facilitate greater design freedom than automated fibre placement (AFP), automated tape laying (ATL) and moulding techniques, particularly regarding fibre placement, localising volume fractions and part geometry.

Several studies have produced novel continuous fibre pathing programs to print unique structures. Hou et al., for example, printed a corrugated structure for use as a core material in composite sandwich paneling, by printing the panel sideways in the Z-direction of the printer [63]. These were reported to exhibit superior compression strengths and lower core densities to those of aluminum corrugated structures. Similarly, Sugiyama et al. printed sandwich structures from carbon fibre; however, these were printed in the XY plane utilising fibre tension to print over the gaps in the core structure [64]. Utilising the continuous fibre filament under tension to form a bridge allows for printing with very little support material. A multitude of core patterns and shapes were tested in 3-point bending, with supporting abutment material being removed using a saw prior to testing. The printing was performed in a single continuous movement with no cutting apparatus present. These studies utilised a layer-by-layer approach for deposition, however a number of studies have taken this a step further to produce 3D curvilinear composites [65,66]. Liu et al. [65] produced a core structure for sandwich paneling, utilising a novel method of depositing small sections of composite in mid-air, without the need for a mould. In this study, the latticed core was printed and then adhesively bonded to epoxy composite face sheets. Compressive strengths were low, however the repeatability and filament placement error were significantly improved during the course of the study. To make a similar curvilinear component, Tse et al. [66] utilised a spring-loaded mechanism to reciprocate a heated nozzle over a 3D printed dissolvable mould. A number of contoured composite parts were produced, and in this case a 2D coordinate system was used to guide the head, with the spring mechanism following the mould in the Z-direction [66]. The exact method of achieving these printing paths is not disclosed in these studies, presumably to prevent replication, however it is likely that each study would have required bespoke programs/scripts to be written.

**Figure 10:**
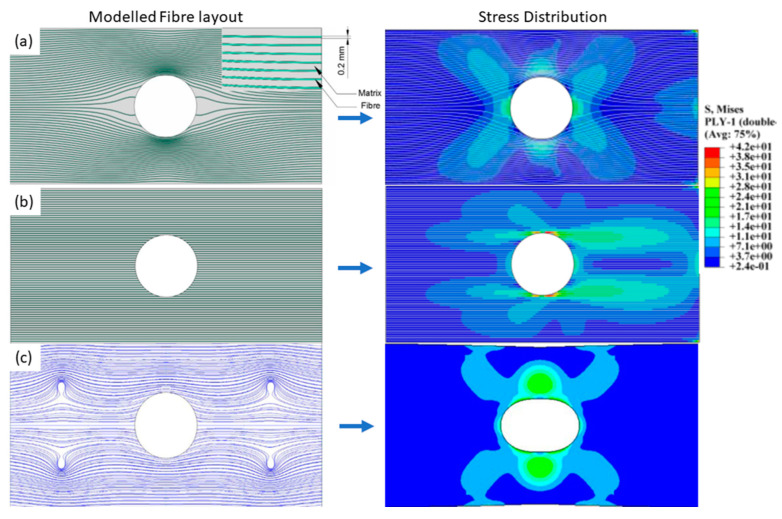
Modelled fibre layouts (left) and resulting stress heat maps (right). (**a**) represents an ideal fibre placement scenario with reduced strain concentration [68]. (**b**) represents a drilled/cut sample with discontinuous fibres resulting in large strain concentrators. (**c**) represents a cut sample containing fibre vorteces to reduce the strain concentration at the centre hole [67].

The use of 3D printing to minimise stress concentrators such as holes and notches has been explored in a number of modelling studies [67,68]. Stress concentrators are a major cause of the early/catastrophic failure of fibre composites. These can be associated, for example, with the machining of holes and notches in conventional composites. 3D printing has the potential to create holes within a part by reorienting fibres around the opening of a hole, rather than breaking the fibres through machining. Yamanaka et al. [67] produced a preliminary model of such a structure, indicating that by 3D printing the composite structure and preventing fibre breakage a significantly increased tensile strength could be achieved, compared with that obtained by the cutting of unidirectional reinforcements (Figure 10c) [67]. This study did not consider the width of a 3D printed fibre filament, and filaments are still cut at the point they reach the perimeter of the hole, meaning that fibre damage was also not accounted for at the point of cutting. Zhang et al. [68] performed a similar study; however, material properties were taken from the 3D printed composite literature, rather than the conventional composite values used by Yamanaka. Laminates were tested in single ply and cross ply configurations. In contrast to the procedure of Yamanaka, the fibres were not cut when the hole perimeter was reached, and instead formed a densified region on either side of the hole perimeter. This densified region prevented a major strain riser formation during testing, increasing the laminates strength and stiffness. Figure 10a,b represent the optimised and cut samples respectively.

Both studies are based upon the simulation of non-woven materials, with precise fibre placement, which are needed in order to reduce the effects of the induced off-axis forces generated by the stress concentration around the hole. Woven materials, however, typically exhibit superior through-thickness and off-axis properties [69]. It is therefore reasonable to conclude that woven laminates would be potentially favourable for resisting the forces developed around a notch.

### 3.4. Six-Axis Robotics in the 3D Printing of Composites

In order to facilitate the wider adoption of 3D composite printing as a manufacturing process, automation, including robotics, is vital. The use of robotic techniques has been utilised for the layup process of composites for a number of years, and most are utilised in combination with ATL systems which, for example, are widely used in the aerospace industry [40]. A multi-axis articulating robot can be used to hold the ATL head, which is in turn connected to a gantry or rail. This can allow 5 to 10 degrees of freedom. This type of system is efficient for the depositing of large non-complex (flat or single curvature) parts; however, for non-flat structures with higher curvature, a second forming process is required after placement. This second forming step has a tendency to cause the warpage and buckling of the laminates, leading to fibre damage [70]. The tape width also precludes tight cornering, and results in machining being required for small feature inclusion.

**Figure 11:**
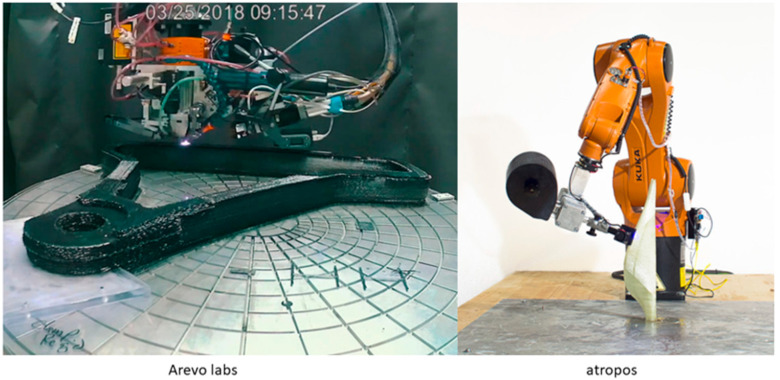
The Arevo labs robotic AM system printing a portion of a bicycle frame (**left**) [71]. The Atropos Robot system printing a glass/epoxy turbine blade without a mould for support (**right**) [72].

Many companies have incorporated robotics as part of their composite printing activities, with a wider range of designs made possible by multi-axis printing. Arevo Labs, for example, reported on the printing of short fibre composites as early as 2016, and has since unveiled a new robotic printing system for the printing of PEEK/CF composites [71]. Arevo have also produced a demonstrator of their technology in the form of a 3D printed CF bicycle frame [71]; this frame is made of layered CF filaments that are deposited using a custom-built, laser-heated deposition head, the details of which are not disclosed (Figure 11). 9T labs have demonstrated a robotic system for the placing of CF/PA12 composite onto curved surfaces, however the details of this design have also not been disclosed publicly [62]. Atropos is a robotic technology demonstrator that originated at the Politechnico Milano +Lab, and features a unique thermosetting resin print head [72]. This system is reported to utilise carbon, glass, or basalt fibres, and can use a number of UV curable resins to produce complex geometric parts (Figure 11). The processing efficiencies obtained through the further automation of FFF composite printing are clearly key for the wider adoption of this 3D printing technology for the manufacture of individualised consumer products, such as sports bicycles, tennis rackets and golf clubs, as well as medical devices such as prostheses.

## 4. Summary and Conclusions

This paper provided an overview of the use of the fused filament fabrication (FFF) technique for the manufacture of both short and continuous fibre-reinforced polymeric materials, along with details of the mechanical properties of the resulting composites. The most widely reported short fibres in the literature are those of carbon and glass fibres, which is primarily due to the application focus within aerospace and automotive. Several other reinforcement fibres, including basalt, aramid and jute (and other natural fibres), are also reported, and have also been shown to improve the mechanical properties of polymer composites. The addition of short fibres into neat thermoplastics polymers can significantly improve their stiffness and strength. However, the maximum achievable properties of these composites are limited due to the presence of porosity in the printed parts. Despite the identified mechanical limitations, the FFF printing of short fibre-reinforced thermoplastic composites shows potential in 3D printing to produce some “end-use” components, such as moulds for tooling. Whilst short fibre composites have excellent utility, and can be processed through a standard FFF process, continuous fibre composites offer orders of magnitude higher strength and stiffness, versus neat polymers or short fibre composites. These materials contain continuous unbroken strands of reinforcing fibre, which allows for greater load capacity, but also requires specialist hardware for processing, such as cutting devices and multi-input printheads for proper polymer–fibre infusion. Two primary methods of continuous composite printing have been highlighted. “In-situ fusion” shows great promise for the rapid manufacturing of composite parts, with potential to produce variable volume fraction parts with a single manufacturing process. This technique, however, typically produces inferior quality parts due to entrained air contents and poor polymer permeation. “Ex-situ prepreg” provides superior quality parts with lower air contents and excellent polymer infusion, at the expense of a more complex multi-stage manufacturing process. A difficulty with the resulting 3D printed composites, however, is the presence of porosity, which can significantly impact on mechanical performance. Amongst the methods of addressing this are the application of pressure during printing, fibre bundle overlapping, the use of low-pressure printing conditions, as well as the use of atmospheric plasma pre-treatments.

The wider adoption of 3D printing for consumer products is facilitated through the use of robotic printing techniques. Developments in this area were reviewed and, when combined with the superior material properties of the high strength and low weight of the 3D printed composites, have the potential to produce a wide range of individualised parts, particularly for sectors such as sports goods and medical devices.

## Figures and Tables

**Table 1:** A summary of materials used for 3D printing of short fibre-reinforced polymer composites.

Matrix	Reinforcement	wt %	Studying	Ref.
PLA	Carbon	15	Experimental characterisation and micrography of 3D printed PLA and PLA reinforced with short carbon fibres.	[26]
ABS	Carbon	5	Effects of process parameters on the tensile properties of FDM fabricated carbon fibre composite parts.	[27]
ABS	Carbon Graphite	5 5	Investigate the effects of reinforcements on porosities and tensile properties of FFF-fabricated parts built at two raster angles.	[28]
PEEK	Carbon Glass	5–15 5–15	Explore the potential engineering application of FDM-3D printing short fibre-reinforced PEEK composites	[29]
PLA	Basalt		Analytical study of the 3D printed structure and mechanical properties of basalt fibre-reinforced PLA composites using X-ray microscopy.	[30]
ABS	Basalt	0–60	Development and mechanical properties of fibre-reinforced ABS for in-space manufacturing applications.	[31]
ABS	Glass	10–18	Study of short fibre-reinforced ABS polymers for use as FFF feedstock material.	[32]
PP	Glass	30	Investigate the effects of fibre reinforcements on the physical and mechanical properties of FFF-fabricated parts.	[33]
